# Osteogenic Differentiation of Human Adipose Tissue-Derived MSCs by Non-Toxic Calcium Poly(ethylene phosphate)s

**DOI:** 10.3390/ijms20246242

**Published:** 2019-12-11

**Authors:** Ilya Nifant’ev, Tatiana Bukharova, Alexander Dyakonov, Dmitry Goldshtein, Elena Galitsyna, Maxim Kosarev, Andrey Shlyakhtin, Dmitry Gavrilov, Pavel Ivchenko

**Affiliations:** 1 Chemistry Department, M.V. Lomonosov Moscow State University, 1–3 Leninskie Gory, 119991 Moscow, Russia; komrad.kosarev.maksim@gmail.com (M.K.); shlyahtinav@mail.ru (A.S.); gavrosdm@gmail.com (D.G.); phpasha1@yandex.ru (P.I.); 2 A.V. Topchiev Institute of Petrochemical Synthesis RAS, 29 Leninsky Pr., 119991 Moscow, Russia; 3 Research Centre for Medical Genetics, 1 Moskvorechye Str., 115522 Moscow, Russia; bukharova-rmt@yandex.ru (T.B.); elemental190@gmail.com (A.D.); dv@rm7.ru (D.G.); snowbars888@yandex.ru (E.G.)

**Keywords:** ring-opening polymerization, polyphosphates, calcium, stem cells, osteoinductivity, osteogenic differentiation, tert-butyl ethylene phosphate, phosphoric acid

## Abstract

There is a current clinical need for the development of bone void fillers and bioactive bone graft substitutes. The use of mesenchymal stem cells (MSCs) that are seeded into 3D scaffolds and induce bone generation in the event of MSCs osteogenic differentiation is highly promising. Since calcium ions and phosphates promote the osteogenic differentiation of MSCs, the use of the calcium complexes of phosphate-containing polymers is highly prospective in the development of osteogenic scaffolds. Calcium poly(ethylene phosphate)s (PEP-Ca) appear to be potentially suitable candidates primarily because of PEP’s biodegradability. In a series of experiments with human adipose-tissue-derived multipotent mesenchymal stem cells (ADSCs), we demonstrated that PEP-Ca are non-toxic and give rise to osteogenesis gene marker, bone morphogenetic protein 2 (*BMP-2*) and mineralization of the intercellular matrix. Owing to the synthetic availability of poly(ethylene phosphoric acid) block copolymers, these results hold out the possibility for the development of promising new polymer composites for orthopaedic and maxillofacial surgery.

## 1. Introduction

Every year, millions of surgical procedures involving bone excision, bone grafting and fracture repair are performed worldwide [1]. The rate of repair after these procedures is dependent on bone defect size and patient age. The lack of the healing capacity of osteogenic tissue results in the prepotency of the fibrous connective tissue, which is de facto a clinical failure [2]. Methods of tissue engineering reflect new insights in chemistry, biology and medicine. These methods aim to construct biological substitutes that can restore and maintain normal function in injured and diseased bone [3].

One of the most promising approaches involves the use of mesenchymal stem cells (MSCs) that are seeded into 3D scaffolds and induce bone generation by osteoinductive cues [1,4,5,6,7]. The design of such 3D scaffolds as a bone void filler can be implemented at the interface of organic chemistry, polymer chemistry, material science and cell biology by the development of the composites containing biodegradable synthetic polymers and calcium phosphates [8,9,10,11]. This approach seems to be prospective due to the variability of polymer characteristics [12,13,14,15] and osteoinductivity of calcium and phosphate ions [16,17]. However, this kind of composite shares some of the challenges arising from the incompatibility of organic polymers with calcium phosphates, therefore resulting in unpredictability of the composite’s behaviour during regeneration against the backdrop of polymer degradation.

**Scheme 1:**
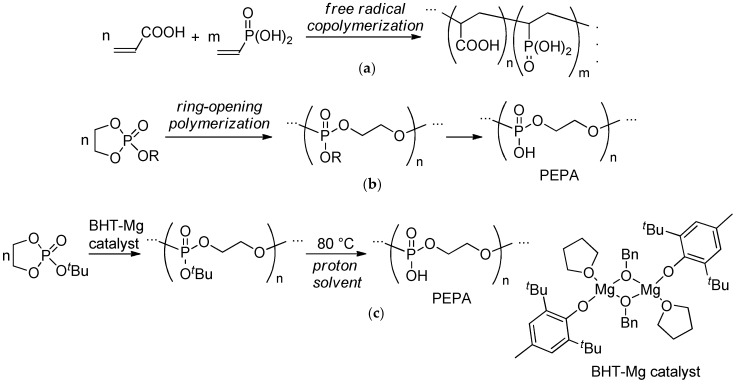
Phosphate-containing polymers: (**a**) Type 1, the products of the radical (co)polymerization of vinyl phosphates; (**b**) Type 2, poly(ethylene phosphoric acid) (PEPA) obtained by ring-opening polymerization (ROP) of cyclic ethylene phosphates followed by hydrolysis; (**c**) Efficient method of PEPA preparation by mild thermolysis of the polymers obtained by the ROP of *tert*-butyl ethylene phosphate (*^t^*BuOEP) [33].

A whole new approach involves the use of synthetic polymers containing phosphate fragments able to chemically bond with calcium ions. Such polymers can be prepared by radical copolymerization involving phosphate-substituted vinyl monomers (Scheme 1a, polymer Type 1) [18,19]. However, in the strict sense, these polymers are not biodegradable. The second type of these prospective polymers can be prepared by the ring-opening polymerization (ROP) of cyclic ethylene phosphate monomers [20,21,22,23,24] followed by the transformation of poly(ethylene phosphate)s to poly(ethylene phosphoric acid)s (PEPA) or their salts [25,26,27,28,29,30,31] (Scheme 1b). The mild thermolysis of the polymers of *tert*-butyl ethylene phosphate (*^t^*BuOEP) was found to be a simple and efficient method of PEPA preparation (Scheme 1c) [32,33]. 

As previously demonstrated [34], the complexes of poly(vinyl phosphate)s with calcium ions show an osteoinductive effect that promotes human bone-marrow-derived MSCs. Despite the obvious promise of poly(ethylene phosphate)s and PEPA for biomedical applications [20,21,22,30,35,36,37,38,39,40,41,42,43,44], the key properties of PEPA calcium complexes—namely, toxicity and ability to provoke MSCs osteogenic differentiation—have not been investigated. In the present communication, we report that such PEPA complexes are non-toxic and give rise to osteogenesis gene marker bone morphogenetic protein 2 (*BMP-2*) and mineralization of the intercellular matrix in experiments with human adipose-tissue-derived multipotent MSCs (ADSCs).

## 2. Results and Discussion

### 2.1. Preparation of PEPA and PEPA Salts

The sample of poly(*^t^*BuOEP) was prepared by the ROP of *^t^*BuOEP in the presence of a heteroleptic complex [(ВНТ)Mg(μ-OBn)(THF)]_2_ as shown in Scheme 1c (see Section 3.1 for details). The composition of the polymer was established by end-group analysis of the ^1^H NMR spectrum as BnO(*^t^*BuOEP)_43_H (*M_n_* = 7.85 × 10^3^, see Figure S1 in the Supporting Information). This composition was in good agreement with SEC data (*M_n_* = 7.91 × 10^3^, *Ð_M_* = 1.24). DSC and TGA demonstrated that poly(*^t^*BuOEP) decomposed at 105–110 °C in bulk [33]. The constant weight of the polymer sample was reached after 6 h of drying at 50 °C and 0.01 Torr.

The solution of PEPA containing the mixture of BnO(HOEP)_43_H and HO(HOEP)_43_H was obtained by heating the aqueous dispersion of BnO(*^t^*BuOEP)_43_H for 40 min at 80 °C. The molar concentration of phosphate groups in the PEPA solution was determined by ^31^P NMR spectroscopy using trimethyl phosphate as an internal standard (see Section 3.1 for details).

Aqueous PEPA solution with a concentration of phosphate groups of 0.443 mmol/g was used for the preparation of sodium and calcium salts by the reactions with calculated amounts of NaHCO_3_ and CaCO_3_ in phosphate/metal molar ratios of 1:1 (**Na**-PEP, **Ca1**-PEP) and 2:1 (**Ca2**-PEP) (see Section 3.1 for details).

### 2.2. The Influence of PEPA Salts on ADSCs’ Adhesion and Proliferation

**Figure 1:**
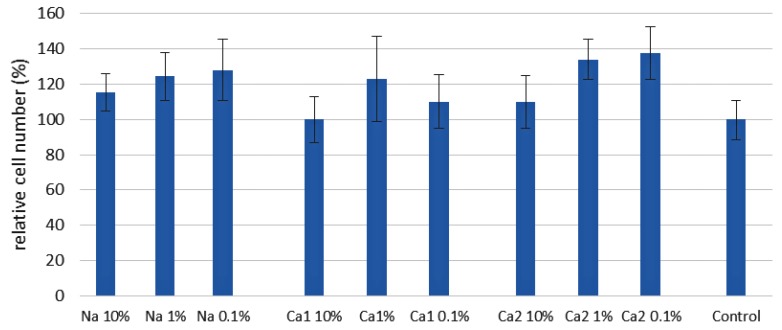
The results of 7-day cell adhesion and proliferation experiments for the solutions of PEPA metal salts diluted by the factors of 1000, 100 and 10. The starting solutions of the salts were prepared in phosphate/metal molar ratios of 1:1 (**Na**-PEP, **Ca1**-PEP) and 2:1 (**Ca2**-PEP). The initial concentration of phosphate groups was 0.443 mmol/g. The percentage of the cell viability relative to control (additive-free plates) is presented.

The analysis of cell adhesion demonstrated that **Ca2**-PEP substantially contributed to the adhesion of ADSCs (see Section 3.3 and Figure S15 in the Supporting Information for details). At the same time, the effect of cell proliferation was insignificantly affected by the solutions of PEPA salts (Figure 1). The major finding from the evaluation of the experimental results was the absence of the toxic effect of PEPA salts. This result is essential for the prospects of biomedical applications of PEPA-containing polymers.

### 2.3. Osteogenic Potential of the PEPA Salts

To examine the effect of PEPA salts on osteogenic induction, ADSCs were cultured in media with PEPA salts of different concentrations ranging from 0.1% to 10%. The results of osteogenic differentiation were determined by the expression level of the *BMP-2* gene using RT-PCR analysis and by mineralization assay with alizarin red S staining (see Section 3.4 for details).

**Figure 2:**
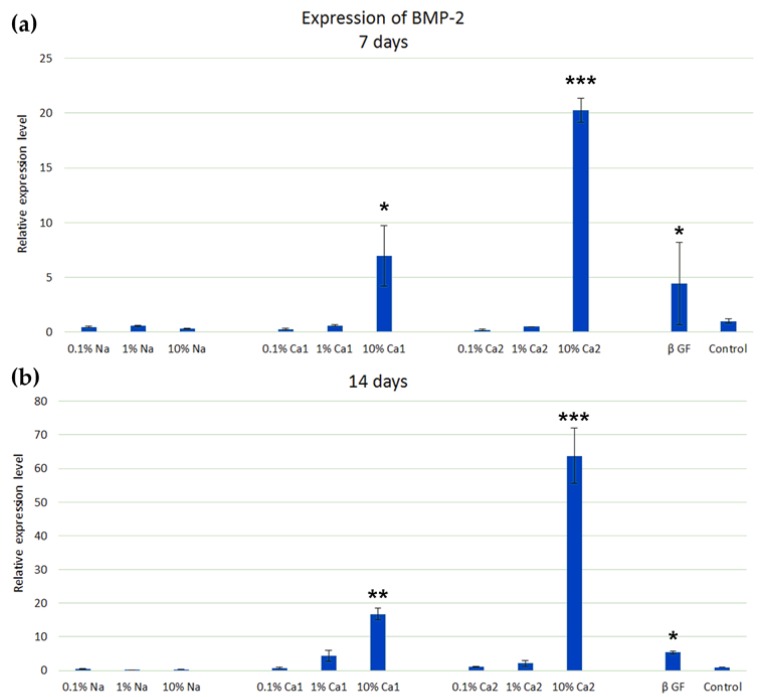
Differentiation of adipose-tissue-derived multipotent mesenchymal stem cells (ADSCs) with the solutions of PEPA salts **Na**-PEP, **Ca1**-PEP and **Ca2**-PEP of different concentrations in the presence of β-glycerophosphate and in the control experiment in the absence of additives. The expression of the *BMP-2* gene in ADSCs on days 7 (**a**) and 14 (**b**). RT-PCR analysis. Statistical difference between a test group and control (* *p* < 0.05, ** *p* < 0.01, *** *p* < 0.001).

**Figure 3:**
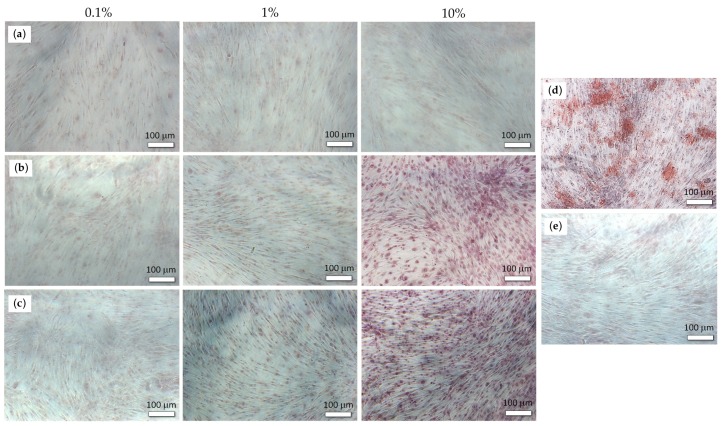
The mineralization of the extracellular matrix of ADSCs on day 14 in the presence of **Na**-PEP (**a**), **Ca1**-PEP (**b**), **Ca2**-PEP (**c**), β-glycerophosphate (**d**) and control experiment in the absence of additives (**e**). The level of Alizarin red S staining.

Our results demonstrated that calcium salts **Ca1**-PEP and **Ca2**-PEP at a concentration of 10% significantly increased the expression of the *BMP-2* gene at 7 and 14 days. The osteogenic effect of PEPA calcium salts was higher than of β-glycerophosphate (Figure 2). We also found significant mineralization of the extracellular matrix during the cultivation of ADSCs with **Ca1**-PEP and **Ca2**-PEP at a concentration of 10% (Figure 3). **Na**-PEP did not affect differentiation of ADSCs.

## 3. Materials and Methods 

### 3.1. Synthesis of PEPA and Metal Salts

#### 3.1.1. General Experimental Remarks

All the synthetic and polymerization experiments were conducted under an argon atmosphere. Tetrahydrofuran (THF), diethyl ether (Et_2_O) and triethylamine were refluxed with Na/benzophenone/dibenzo-18-crown-6 and distilled prior to use. Pentane was refluxed for 10 h over sodium and then distilled and stored under an argon atmosphere over sodium. 2,6-Di-*tert*-butyl-4-methylphenol (BHT, ≥99%, Merck, NJ, USA), di-*n*-butylmagnesium (1.0 M solution in heptane, Merck, NJ, USA) and acetic acid (≥99.9%, Acros Organics, Geel, Belgium) were used as purchased. Benzyl alcohol (BnOH, 99%, Acros Organics, Geel, Belgium) and *tert*-butyl alcohol (*^t^*BuOH, 99%, Merck, NJ, USA) were distilled over BaO and stored under argon. 2-Chloro-2-oxo-1,3,2-dioxaphospholane [45], 2-*tert*-butoxy-2-oxo-1,3,2-dioxaphospholane *^t^*BuOEP [23], and BHT-Mg catalyst [(ВНТ)Mg(μ-OBn)(THF)]_2_ [46] were prepared according to previously described methods (for details, see Section S1 in the Supporting Information).

#### 3.1.2. Synthesis of Poly(^t^BuOEP) 

The solution of the catalyst [(ВНТ)Mg(μ-OBn)(THF)]_2_ (125 mg, 0.296 mmol) in CH_2_Cl_2_ (4.1 mL) was introduced to the vial containing *^t^*BuOEP (2.058 g, 11.424 mmol) at 20 °C. After 70 h, the polymer was precipitated from the reaction mixture by the addition of pentane (25 mL). The polymer was separated, dissolved in 5 mL of CH_2_Cl_2_ and passed through a 0.45 µm PTFE membrane filter using a syringe. The solvents were removed under reduced pressure, and the residue was dried at 50 °C and 0.01 Torr to constant weight. The yield was 1.64 g (80%) of pale-yellow sticky solid, *M_n_* = 7.91 × 10^3^, *Ð_M_* = 1.24 (SEC). For ^1^H and ^31^P NMR spectra of the polymer obtained, see Figures S9 and S10 in the Supporting Information.

#### 3.1.3. Preparation of the PEPA Solution

Poly(*^t^*BuOEP) (15,729 g) was dispersed in distilled water (14.3 g). The emulsion was heated with stirring at 80 °C for 40 min. After heating, the solution was washed by CH_2_Cl_2_ (4 × 10 mL), heated to 60 °C for CH_2_Cl_2_ removal, degassed under vacuum, and passed through a 0.45 µm PTFE membrane filter. The filter was washed by a minimal volume of water. As a result, 19.7 g of PEPA solution was obtained. The molar concentration of phosphate groups in PEPA solution was determined as 0.443 M by ^31^P NMR spectroscopy using trimethyl phosphate as an internal standard (see Figure S13 in the Supporting Information).

#### 3.1.4. Preparation of the Solutions of PEPA Metal Salts

**Na**-PEP. NaHCO_3_ (223 mg, 2.66 mmol) was added to PEPA stock solution (6 g, 2.66 mmol of P(O)OH groups). After the cessation of CO_2_ evaluation, the solution was passed through a 0.45 µm PTFE membrane filter. **Ca1**-PEP and **Ca2**-PEP were obtained in a similar way by the reactions of СaCO_3_ (266 mg/2.66 mmol or 133 mg/1.33 mmol, respectively) with PEPA (6 g, 2.66 mmol of P(O)OH groups).

### 3.2. Cultivation of the ADSCs

Based on the data compiled and reported earlier [47,48,49], human adipose-tissue-derived stem cells (ADSCs) were isolated from lipoaspirate obtained by liposuction after approval from the Research Centre for Medical Genetics (RCMG) Ethics Committee (The permit to use the cell cultures in research was granted by Bioethics Committee of the RCMG, Record No. 6/5, approved on 15 November 2016). ADSC was cultured in Dulbecco's Modified Eagle Medium F-12 (DMEM-F12) (PanEco, Moscow, Russian Federation) containing 10% foetal bovine serum (FBS; PAA Laboratories, Linz, Austria), 4 mM L-glutamine (PanEco, Moscow, Russian Federation) and 0.1 mg/mL amikacin (Joint-Stock Company Kurgan Medicines and Products Sintez, Kurgan, Russian Federation) at 37 °C in a 5% CO_2_ humidified atmosphere.

### 3.3. The Study of the Influence of PEPA Salts on Cell Adhesion and Proliferation

For the study of the influence of PEPA salts on cell adhesion and proliferation, PEPA salts with a concentration of 0.443 mmol/g were dissolved in a physiological solution (PanEco, Moscow, Russian Federation) to concentrations of 0.1%, 1% and 10%. PEPA salt solutions were applied to the wells of a 48-well 300 μL culture plate for 2 h at 37 °C in order to adsorb PEPA salt onto the plates. Then, unbound PEPA salt was removed. Control plates were salt free.

ADSC cells were seeded to the surface of the PEPA-adsorbed 48-well culture plates at a density of 2.0 × 10^4^ cells/well and cultured in growth medium at 37 °C in a 5% CO_2_ humidified atmosphere. The number of cells were counted in six to ten randomly selected fields in each well after one and seven days. To visualize each individual cell, the nuclei were stained with 4′,6-diamidino-2-phenylindole (DAPI) (Sigma-Aldrich, St. Louis, MO, USA), 1 µg/mL in PBS (see Figure S14 in the Supporting Information).

### 3.4. The Study of the Osteogenic Potential of PEPA Salts

For osteogenic differentiation, ADSCs were cultured in 48-well culture plates at a density of 2.0 × 10^4^ cells per well in Dulbecco's Modified Eagle Medium (DMEM) (PanEco, Moscow, Russian Federation) containing 10% FBS (PAA Laboratories, Linz, Austria), 50 mg/L l-ascorbic acid (Sigma-Aldrich, St. Louis, USA), 4 mM l-glutamine (PanEco, Moscow, Russian Federation), 0.1 mg/mL amikacin (Joint-Stock Company Kurgan Medicines and Products Sintez, Kurgan, Russian Federation) and PEPA salts at concentrations of 0.1%, 1% and 10% or 10 mМ/L β-glycerophosphate (Sigma-Aldrich, St. Louis, MO, USA) at 37 °C in a 5% CO_2_ humidified atmosphere.

In vitro mineralization was analysed 14 days after osteogenic differentiation. The ADSCs were fixed in 70% ethanol (Ferain, Moscow, Russian Federation) for 30 min and stained with 40 mM Alizarin Red S (pH 4.12, PanEco, Moscow, Russian Federation) for 10 min. The plates were washed with distilled water and analysed with an Axio Observer.D1 microscope equipped with AxioCam HRc (Carl Zeiss Ltd., Cambridge, United Kingdom).

For gene expression analysis, total RNA was extracted from cells after differentiation using the RNeasy Mini Kit (QIAGEN, Hilden, Germany) according to the manufacturer’s instructions. cDNA was synthesized using the RevertAid™ First Strand cDNA Synthesis Kit (Thermo Fisher Scientific, Waltham, MS, USA) using reverse transcriptase M-MuLV and oligo-dT primers. A quantitative real-time polymerase chain reaction (RT-PCR) was run in a CFX-96 (Bio-Rad Laboratories, Hercules, CA, USA) PCR thermal cycler three times for each sample using 5×qPCRmix-HS SYBR (Evrogen, Moscow, Russian Federation) and specific primers for osteoblast-related *BMP-2*: forward 5′-ACTACCAGAAACGAGTGGGAA-3′, reverse 5′-GCATCTGTTCTCGGAAAACCT-3′ and glyceraldehyde-3-phosphate dehydrogenase (GAPDH): forward 5′-GAAGGTGAAGGTCGGAGTACA-3′ and reverse 5′-TTCACACCCATGACGAGACAT-3. The RT-PCR was performed as following: denaturation at 95 °C for 6 min followed by 45 cycles of denaturation at 95 °C for 10 s, annealing at 55.6–62.3 °C for 15 s, then elongation at 72 °C for 20 s. The specificity of real-time PCR was verified by melting curve analysis (70–98 °C, with a 0.5 °C increment each cycle). The obtained results of the *BMP-2* expression were normalized by GAPDH expression in the same sample. The relative expression of genes of interest was calculated by the 2^−ΔΔ*C*t^ method.

### 3.5. Statistical Analysis

One-way ANOVA followed by Dunnett’s multiple comparisons test was performed using GraphPad Prism version 7.00 for Windows (GraphPad Software, La Jolla, CA, USA). The values reported are the average ± standard deviations. Values of *p* < 0.05 were considered statistically significant.

## 4. Conclusions

We prepared sodium and calcium salts of poly(ethylene phosphoric acid) (PEPA) and studied the influence of these salts on the growth and differentiation of human adipose tissue-derived stem cells (ADSCs). We found that neither sodium nor calcium PEPA salts demonstrated a toxic effect in 7-day experiments. Furthermore, calcium PEPA salts clearly induced osteogenic differentiation of ADSCs, whereas sodium salts were inactive, within the margin of experimental error. In considering the possibility of the synthesis of polyester–PEPA block copolymers, we can develop next-generation polymer scaffolds for tissue engineering with regards to orthopaedic and maxillofacial surgery.

## Supplementary Materials

Supplementary materials can be found at https://www.mdpi.com/1422-0067/20/24/6242/s1. Detailed description of the synthesis of ^t^BuOEP and BHT-Mg catalyst. NMR spectra of ethylene phosphite and ethylene phosphates (Figures S1–S7), [(ВНТ)Mg(μ-OBn)(THF)]_2_ (Figure S8), poly(^t^BuOEP) (Figures S9 and S10) and PEPA (Figures S11–S13). Figure S14, The microphotograph of DAPI-coloured cells; Figure S15, The results of cell adhesion and proliferation experiments for the solutions of PEPA metal salts (**Na**, **Ca1** and **Ca2**) diluted by the factors of 1000, 100 and 10. The numbers of cells in the field of view are presented in blue (after 1 day) and in red (after 7 days).

## Abbreviations

ADSCs	human adipose-tissue-derived stem cells
BHT	2,6-di-*tert*-butyl-4-methylphenol
BMP-2	bone morphogenetic protein 2
DSC	differential scanning calorimetry
GAPDH	glyceraldehyde-3-phosphate dehydrogenase
MSCs	mesenchymal stem cells
PEP	poly(ethylene phosphate)
PEPA	poly(ethylene phosphoric acid)
PTFE	poly(tetrafluoroethylene)
RCMG	Research Centre of Medical Genetics
ROP	ring-opening polymerization
TGA	thermogravimetric analysis
