# Supplementary material for: Osteogenic Differentiation of Human Adipose Tissue-Derived MSCs by Non-Toxic Calcium Poly(ethylene phosphate)s
Source: Int J Mol Sci. 2019 Dec 11;20(24):6242. doi: 10.3390/ijms20246242 (PMC6940807; doi:10.3390/ijms20246242)
Supplement: Supplementary file 1 [file ijms-20-06242-s001.pdf]

Osteogenic differentiation of human adipose tissue-derived MSCs by non-toxic calcium poly(ethylene phosphate)s

Ilya Nifant'ev [1,2,*], Tatiana Bukharova [3], Alexander Dyakonov [3], Dmitry Goldshtein [3], Elena Galitsyna [3], Maxim Kosarev [1], Andrey Shlyakhtin [1], Dmitry Gavrilov [1] and Pavel Ivchenko [1,2]

[1] Chemistry Department, M.V. Lomonosov Moscow State University, 1–3 Leninskie Gory, 119991 Moscow, Russia; komrad.kosarev.maksim@gmail.com (M.K.); shlyahtinav@mail.ru (A.S.);
phpasha1@yandex.ru (P.I.)

[2] A.V. Topchiev Institute of Petrochemical Synthesis RAS, 29 Leninsky Pr., 119991 Moscow, Russia.

[3] Research Centre for Medical Genetics, 1 Moskvorechye Str., 115522 Moscow, Russia; bukharova-rmt@yandex.ru

* Correspondence: ilnif@yahoo.com or inif@org.chem.msu.ru; Tel.: +7-495-939-4098

SUPPORTING INFORMATION

S1. Synthesis of polymers and metal salts 2
S2. NMR spectra of polymers 8
S3. Study of cell proliferation 11

S1. Synthesis of polymers and metal salts

2-Chloro-1,3,2-dioxaphospholane

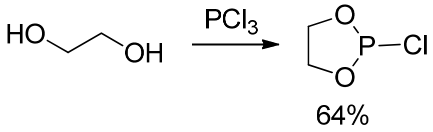

The compound was synthesized via a modified literature protocol [45]. A flame-dried 500 ml three-neck flask, equipped with a dropping funnel and a reflux condenser with a calcium chloride tube, was charged with phosphorous trichloride (137.33 g, 1 mol) in dry dichloromethane (150 ml). Ethylene glycol (62.07 g, 1 mol) was added dropwise to the stirring solution. Argon was bubbled through the solution to remove hydrogen chloride. After 2 h, the solvent was removed and the residue was purified twice by distillation under reduced pressure. The yield was 80.7 g (64%). B. p. 83-84 °C (79-81 Torr), colorless liquid.

^{1}H NMR (400 MHz, $CDCl_3$, 20 °C): δ 4.44 (m, 2H); 4.22 (m, 2H).

^{31}P{H} NMR (162 MHz, $CDCl_3$, 20 °C): δ 167.61.

For NMR spectra, see Figures S1, S2.

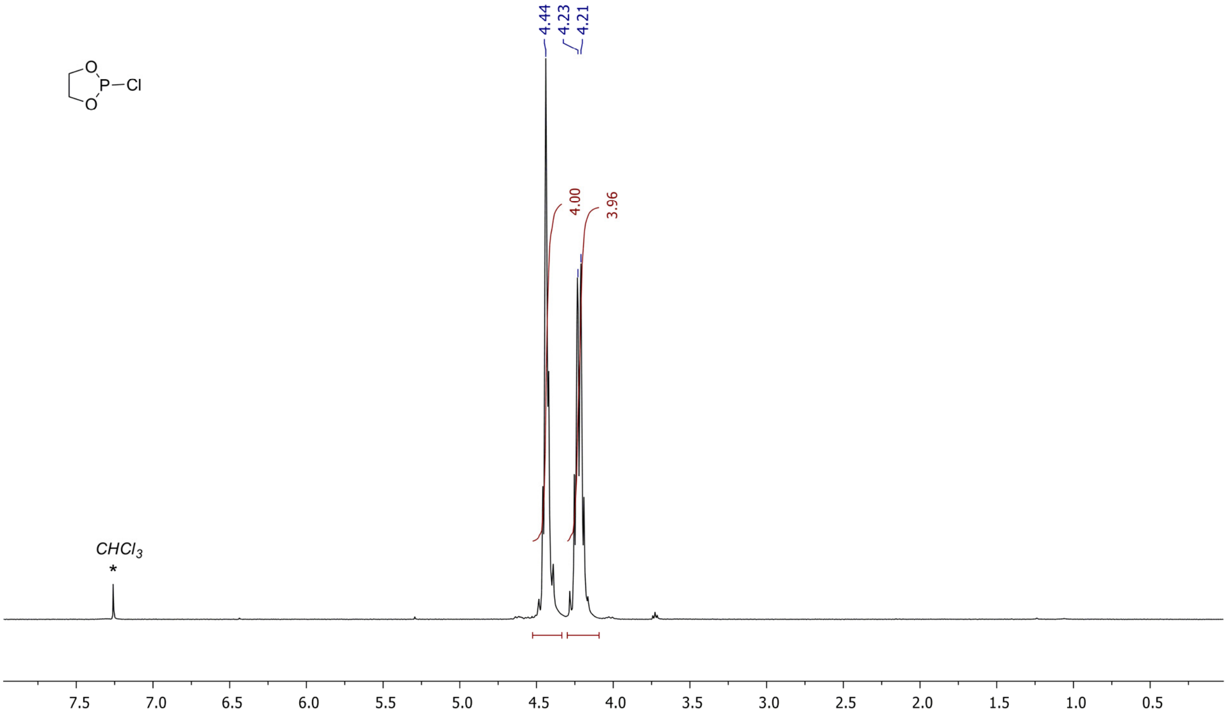

Figure S1. ^{1}H NMR spectrum (400 MHz, $CDCl_3$, 20 °C) of 2-chloro-1,3,2-dioxaphospholane.

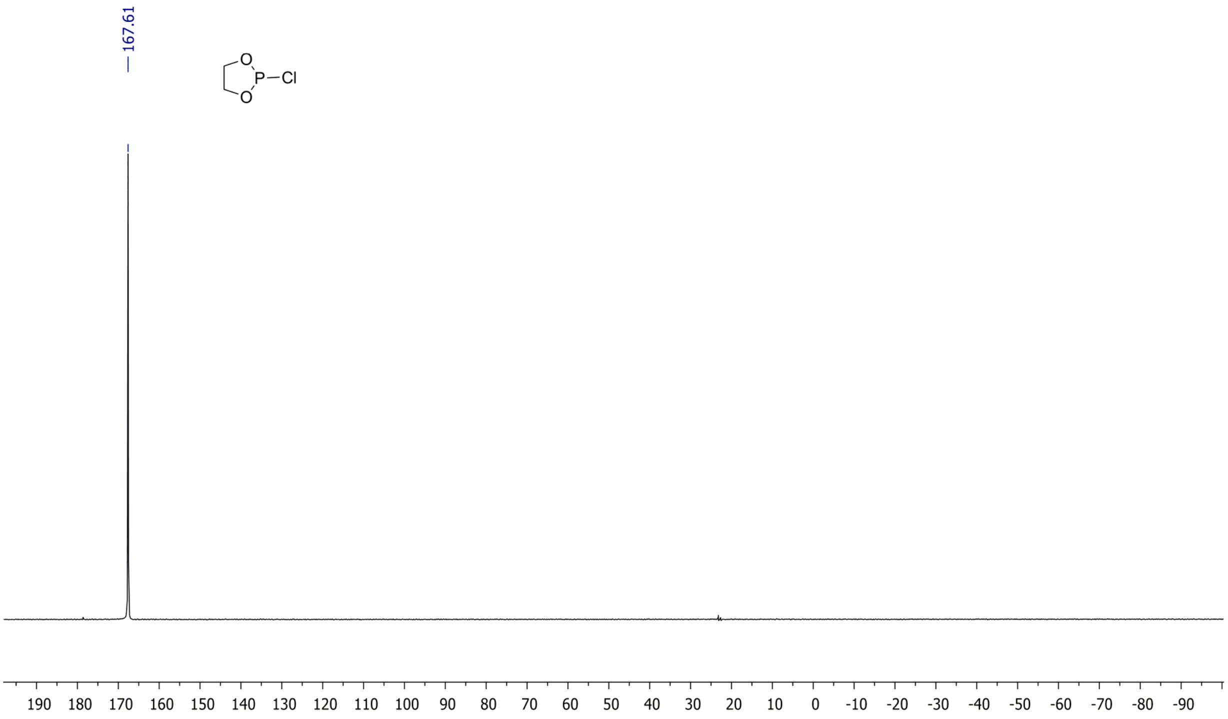

Figure S2. $^{31}P\{H\}$ NMR spectrum (162 MHz, $CDCl_3$, 20 °C) of 2-chloro-1,3,2-dioxaphospholane.

2-Chloro-2-oxo-1,3,2-dioxaphospholane

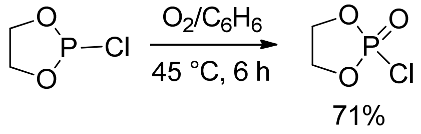

The compound was synthesized according to a modified literature procedure [45]. A flame-dried 500 ml three-neck flask, equipped with a reflux condenser, was charged with 2-chloro-1,3,2-dioxaphospholane (50 g, 0.4 mol), benzene (200 ml) was added, and the mixture was heated to 50 °C. A stream of oxygen was passed through the solution for 12 h. The solvent was removed *in vacuo* and the residue was purified by distillation under reduced pressure. The yield was 40.1 g (71%). B. p. 79-80 °C (0.4 Torr), colorless liquid.

^{1}H NMR (400 MHz, $CDCl_3$, 20 °C): δ 4.63-4.46 (m, 4H).

$^{31}P\{H\}$ NMR (162 MHz, $CDCl_3$, 20 °C): δ 22.81.

For NMR spectra, see Figures S3, S4.

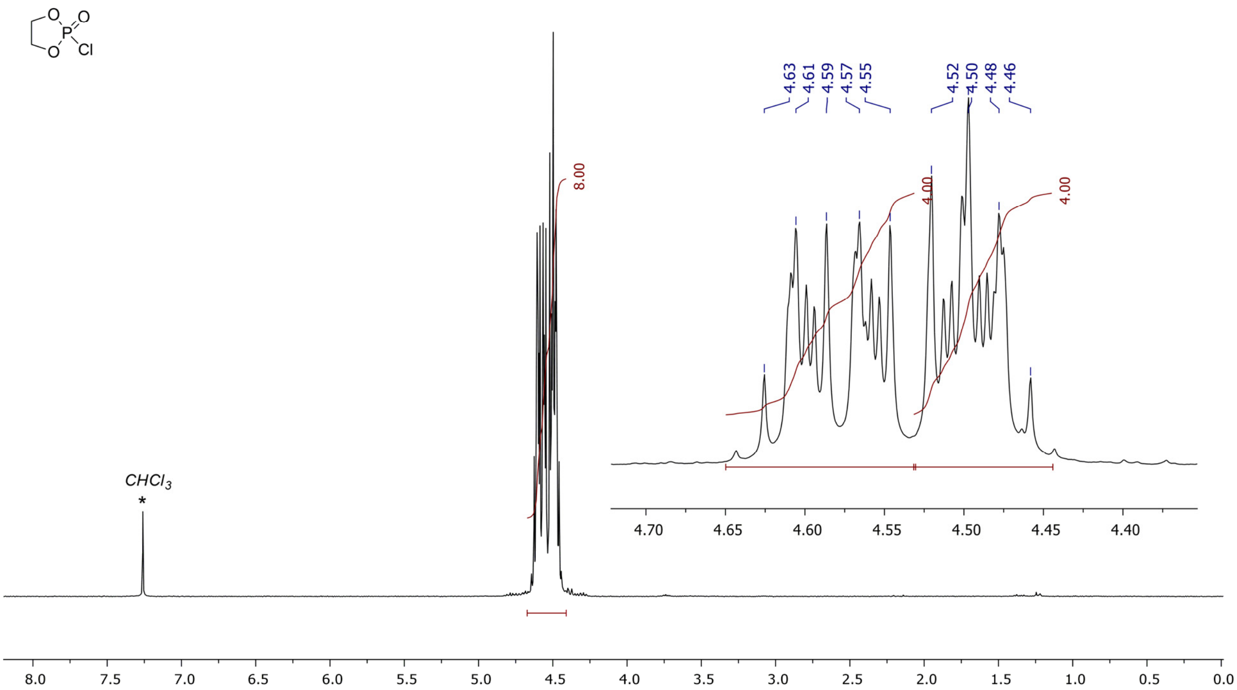

Figure S3. ^{1}H NMR spectrum (400 MHz, $CDCl_3$, 20 °C) of 2-chloro-2-oxo-1,3,2-dioxaphospholane.

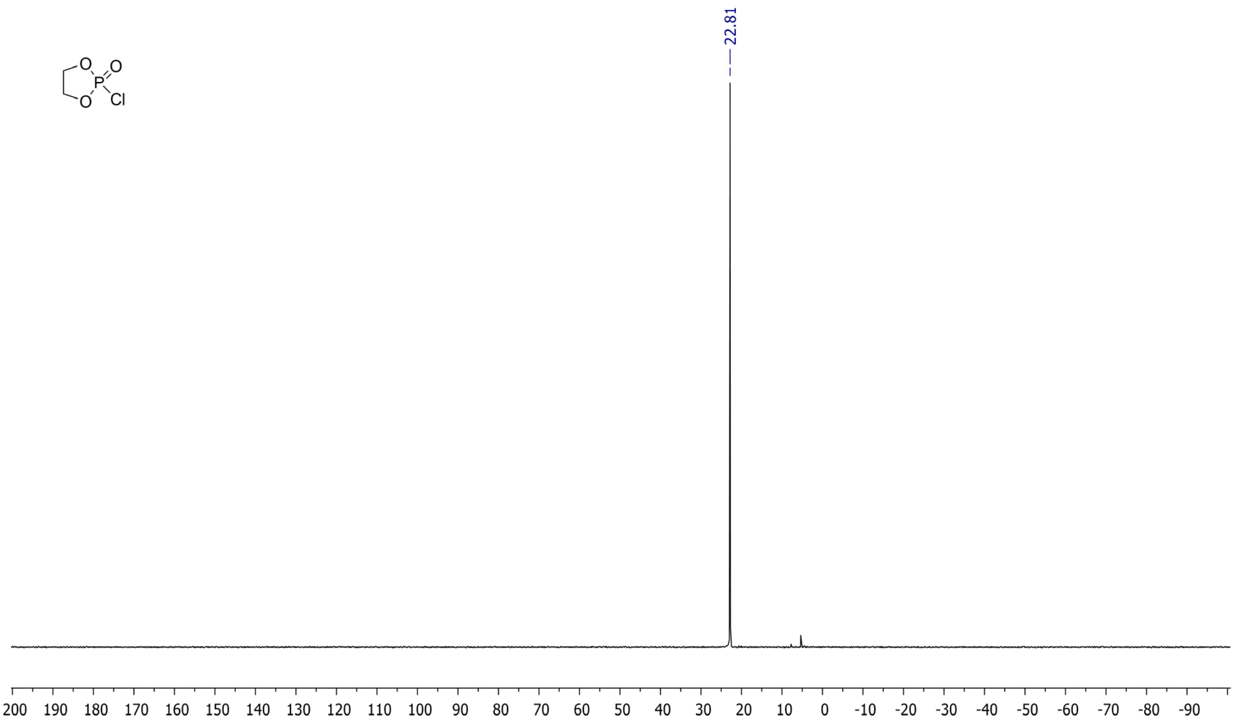

Figure S4. $^{31}P\{^{1}H\}$ NMR spectrum (162 MHz, $CDCl_3$, 20 °C) of 2-chloro-2-oxo-1,3,2-dioxaphospholane.

2-*tert*-Butoxy-2-oxo-1,3,2-dioxaphospholane (*tert*-butyl ethylene phosphate, *t*BuOEP)

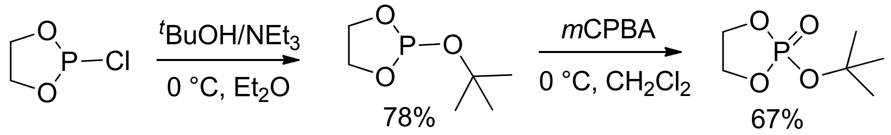

The compound was synthesized via a modified literature protocol [23].

2-tert-Butoxy-1,3,2-dioxaphospholane.

A flame-dried 1000 ml three-neck flask, equipped with a dropping funnel, was charged with dry tert-butanol (38.6 g, 0.52 mol), dry triethylamine (78 ml, 0.52 mol) and dry diethyl ether (500 ml). A solution of 2-chloro-oxo-1,3,2-dioxaphospholane (66.0 g, 0.52 mol) in dry ether (60 ml) was added dropwise under stirring at 0 °C. The mixture was allowed to warm to room temperature and stirred overnight. Triethylammonium chloride was filtered off, and the filtrate was concentrated *in vacuo*. The residue was purified by distillation under reduced pressure. The yield was 67.1 g (78%). B. p. 70-75°C (13 Torr), colorless liquid.

^{1}H NMR (400 MHz, $CDCl_3$, 20 °C): δ 4.14 (m, 2H, $OC\underline{H}_2$); 3.88 (m, 2H, $OC\underline{H}_2$); 1.47 (s, 9H, O-$C(C\underline{H}_3)_3$.

^{31}P{H} NMR (162 MHz, $CDCl_3$, 20 °C): δ 134.57 (s).

2-tert-Butoxy-2-oxo-1,3,2-dioxaphospholane.

A flame-dried 500 ml three-neck flask, equipped with a dropping funnel, was charged with 2-*tert*-butoxy-1,3,2-dioxaphospholane (8.2 g, 0.05 mol) in dry dichloromethane (100 ml). A solution of *m*-chloroperbenzoic acid (~0.055 mol) prepared by drying of the mixture of commercial 70-77% *m*-chloroperbenzoic acid (13 g) in 100 ml of dichloromethane over $MgSO_4$, was added dropwise under stirring at 0 °C within 3 h. The resulting precipitate of *m*-chlorobenzoic acid was removed by filtration. The filtrate was treated with the aq. solutions of 15% K_2CO_3 (2×40 ml) and 20% sodium thiosulfate (2×30 ml), dried over $MgSO_4$ and concentrated *in vacuo* to give white crystalline substance (M. p. 28 °C). The yield was 6.1 g (67%).

^{1}H NMR (400 MHz, $CDCl_3$, 20 °C): δ 4.33 (m, 2H, $OC\underline{H}_2$); 4.26 (m, 2H, $OC\underline{H}_2$); 1.47 (s, 9H, O-$C(C\underline{H}_3)_3$.

^{13}C{H} NMR (101 MHz, $CDCl_3$, 20 °C): δ 84.08 (d, $^2J_{CP}$ = 7.2 Hz, 1C, O-$\underline{C}(CH_3)_3$); 65.52 (d, $^2J_{CP}$ = 2.3 Hz, 2C, $O\underline{C}H_2CH_2O$); 29.67 (d, $^3J_{CP}$ = 4.4 Hz, 3C, O-$C(\underline{C}H_3)_3$).

^{31}P{H} NMR (162 MHz, $CDCl_3$, 20 °C): δ 13.21 (s).

For NMR spectra, see Figures S5–S7.

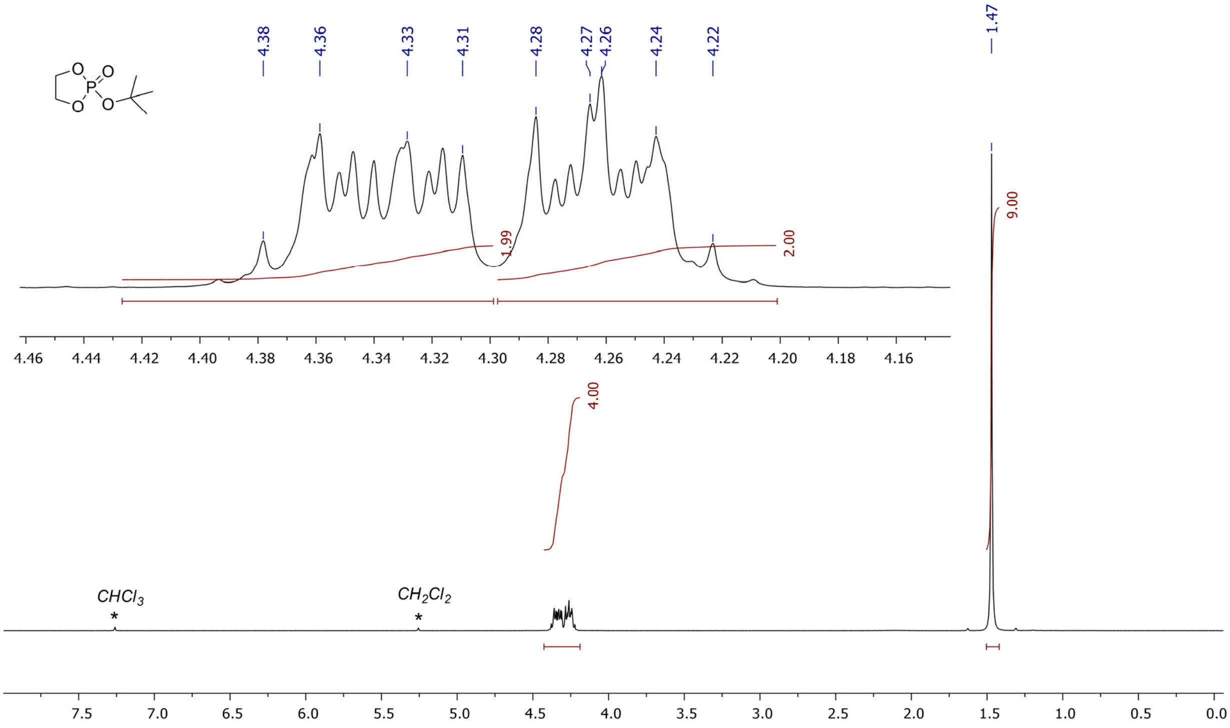

Figure S5. ^{1}H NMR spectrum (400 MHz, $CDCl_3$, 20 °C) of ^{t}BuOEP.

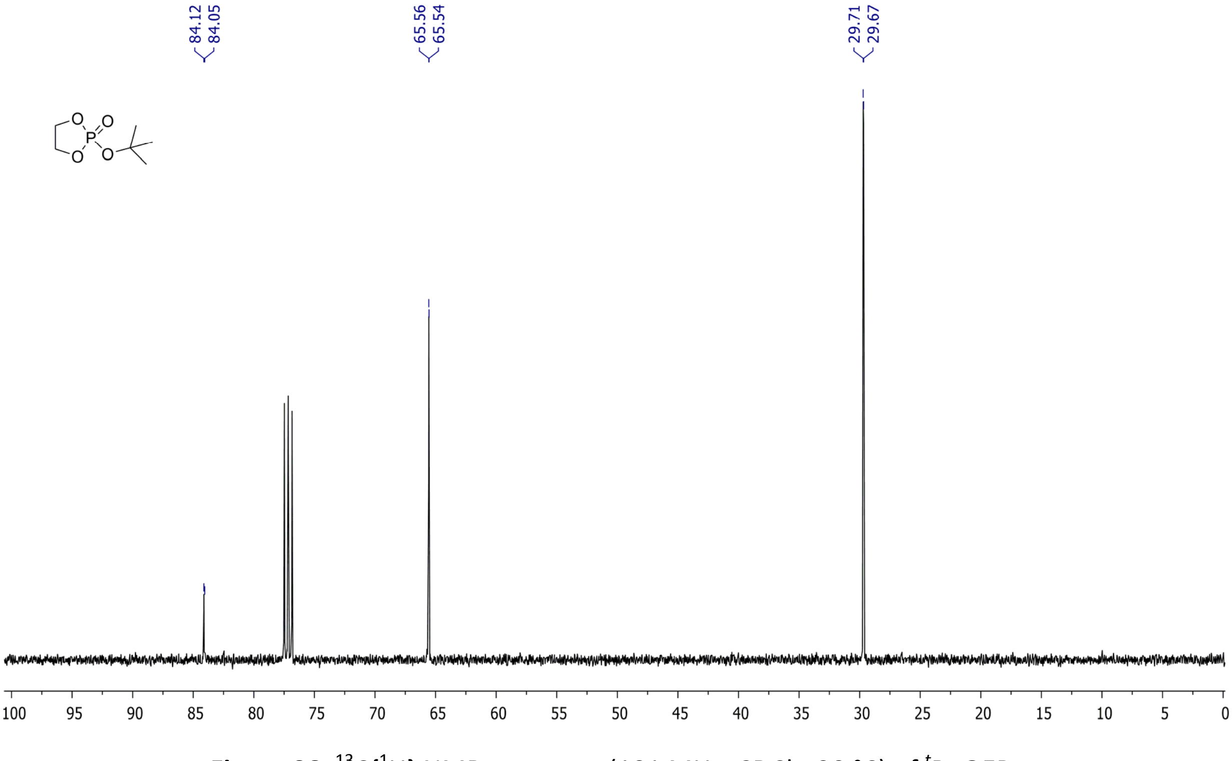

Figure S6. $^{13}C\{^{1}H\}$ NMR spectrum (101 MHz, $CDCl_3$, 20 °C) of ^{t}BuOEP.

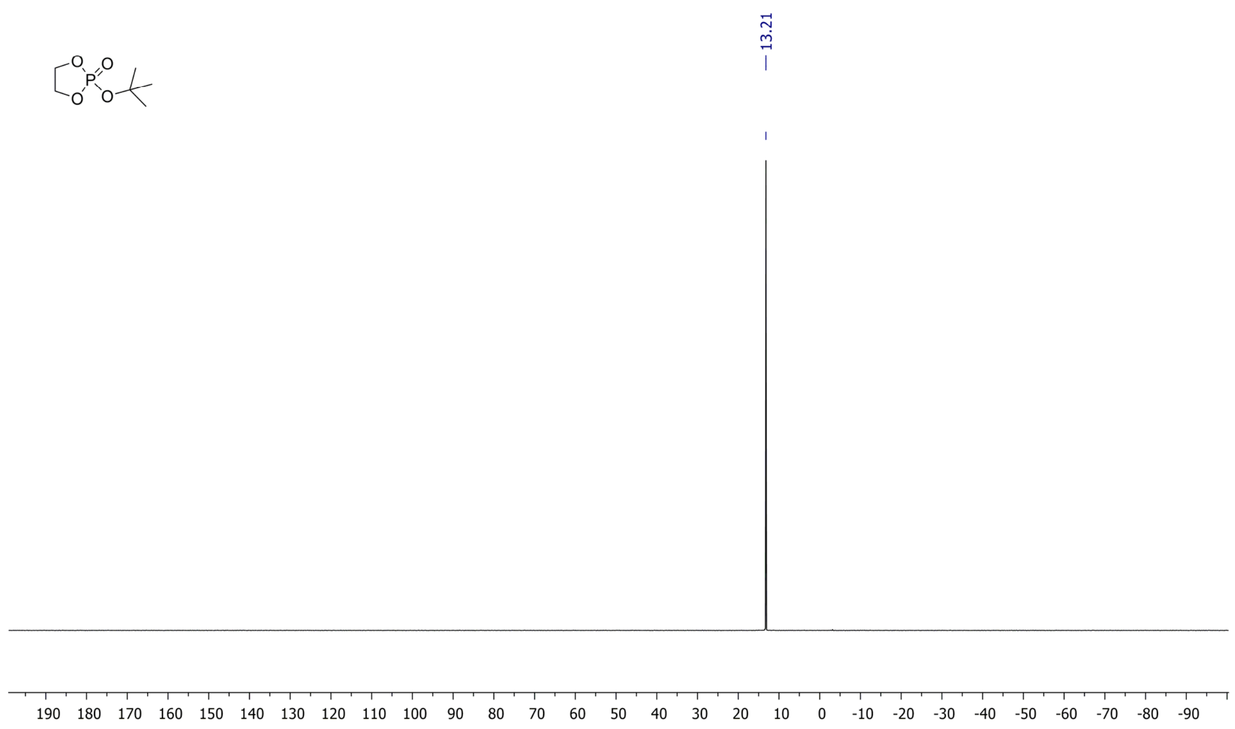

Figure S7. ^{31}P{^{1}H} NMR spectrum (162 MHz, $CDCl_3$, 20 °C) of ^{t}BuOEP.

[(BHT)Mg(μ-BnO)(THF)]$_2$

The compound was synthesized according to a modified literature procedure [46]. A solution Bu_2Mg in heptane (20 ml, 1 M, 20 mmol) was dropwise added to a stirred solution of BHT (4.410 g, 20 mmol) in a toluene/THF mixture (8 ml and 4.5 ml, correspondingly). After 40 min, a solution of BnOH (2.168 g, 20 mmol) in THF (1 ml) was dropwise added to the stirred reaction mixture. The formed solution was then stirred for 3 min. After 5 min, crystals of **4** started to form. Two hours later, the mother liquor was decanted. The crystals were washed with toluene (2×5 ml) and hexane (2×5 ml), dried under dynamic vacuum till the constant weight. The yield was 6.785 g (80%).

^{1}H NMR (400 MHz, THF-d_8, 20 °C): δ 7.37 (d, ^{3}J = 7.7 Hz, 2H, o-H_{Ph}); 7.20 (t, ^{3}J = 7.6 Hz, 2H, m-H_{Ph}); 7.13 (t, ^{3}J = 7.6 Hz, 1H, p-H_{Ph}); 6.77 (s, 2H, m-H_{BHT}); 5.02 (s, 1H, O-C$\underline{H}_2$-Ph); 3.64-3.59 (m, 4H, $CH_2C\underline{H}_2O_{THF}$); 2.13 (s, 3H, -C$\underline{H}_3$ $_{BHT}$); 1.80-1.75 (m, 4H, $C\underline{H}_2CH_2O_{THF}$); 1.37 (s, 18H, 2,6-$^{t}Bu_2$ $_{BHT}$).

^{13}C{^{1}H} NMR (101 MHz, THF-d_8, 20 °C): δ 161.4 (ipso-C-O_{BHT}); 145.8 (ipso-C-CH_2 $_{BnO}$); 137.7 (o-C-^{t}Bu $_{BHT}$); 129.1 (o-C_{BnO}); 128.1 (m-C_{BHT}); 127.6 (p-C_{BnO}); 125.7 (m-C_{BnO}); 121.0 (p-C-Me_{BHT}); 68.4 ($CH_2\underline{C}H_2O_{THF}$); 66.6 (Ph-$\underline{C}H_2$-O); 35.7 (-$\underline{C}Me_3$ $_{BHT}$); 31.4 (-C($\underline{C}H_3)_3$ $_{BHT}$); 26.5 ($\underline{C}H_2CH_2O_{THF}$); 21.6 (p-$CH_3$ $_{BHT}$).

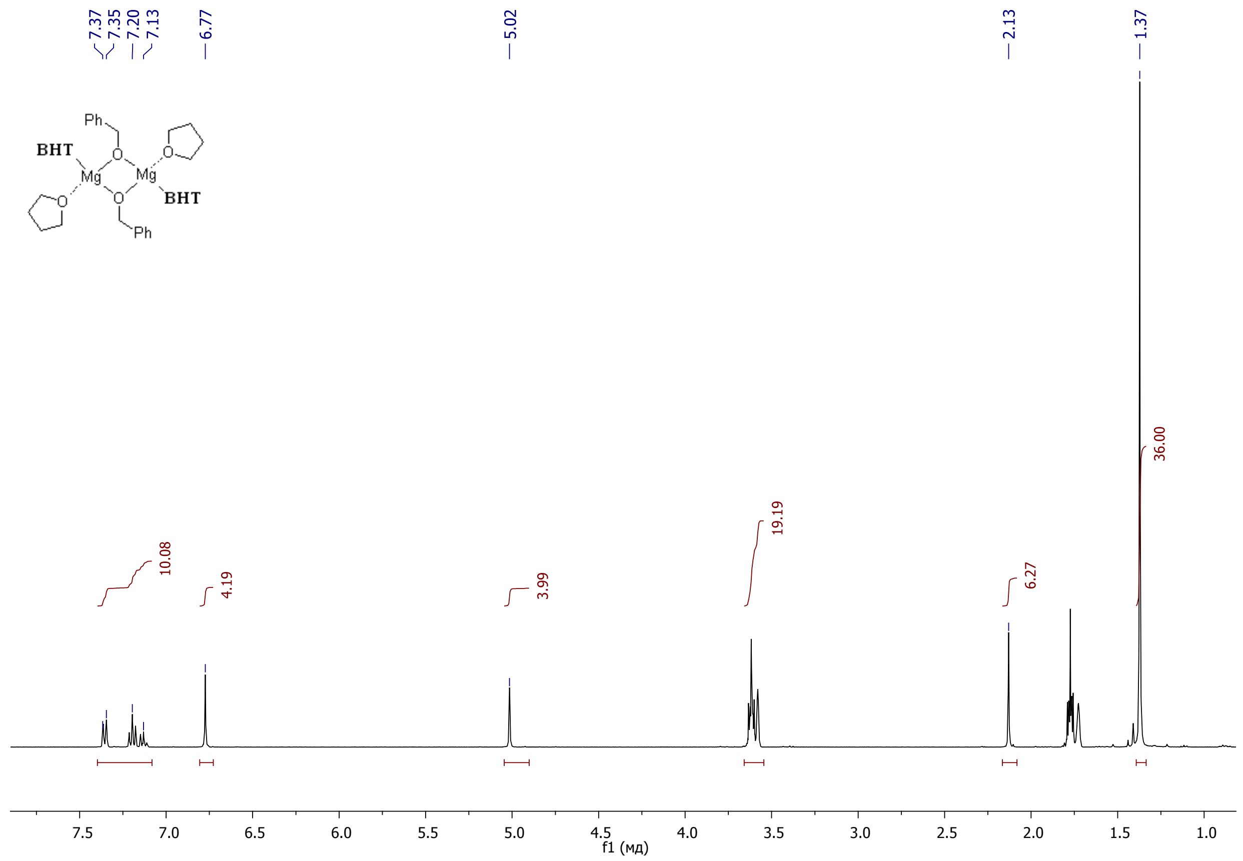

Figure S8. ^{1}H NMR spectrum (400 MHz, THF-d_8, 20 °C) of [(μ-$PhCH_2O$)Mg(BHT)(THF)]$_2$.

S2. NMR spectra of polymers

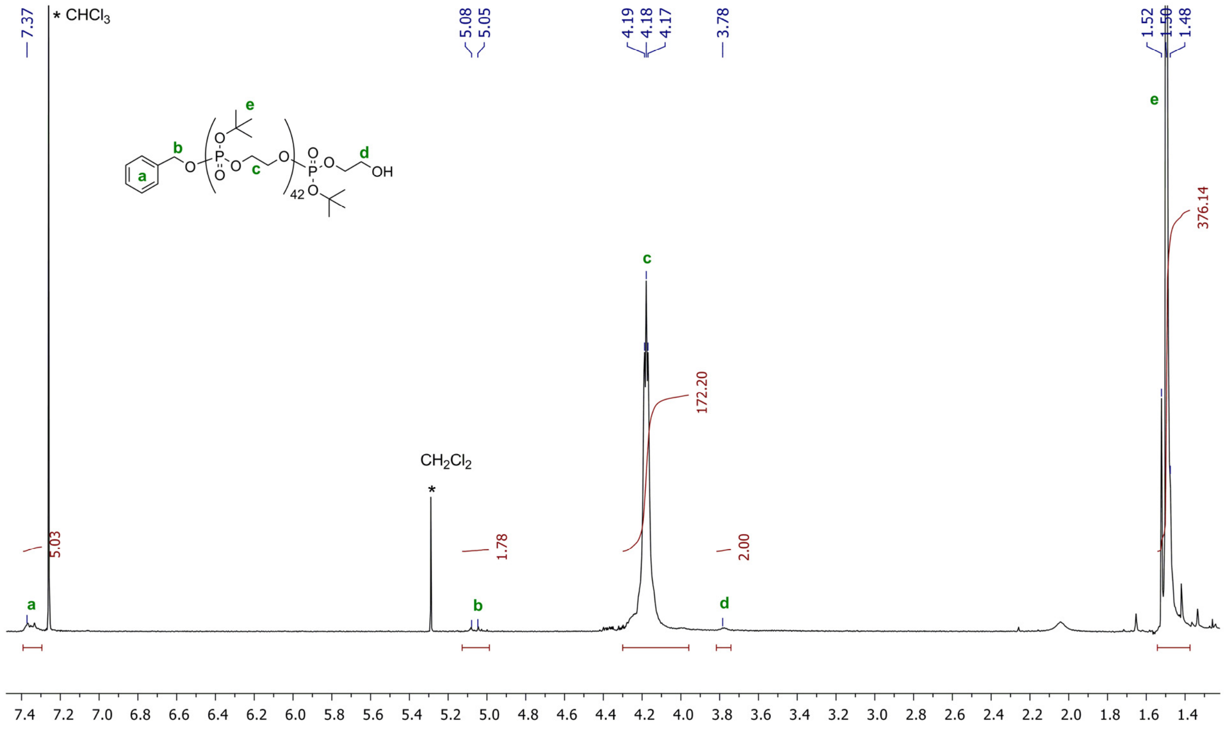

Figure S9. ^{1}H NMR spectrum (400 MHz, $CDCl_3$, 20 °C) of poly(^{t}BuOEP). The signals of Bn end group (a, b), ethylene phosphate fragment (c), CH_2OH end-group (d) and *tert*-butyl fragment (e) are highlighted.

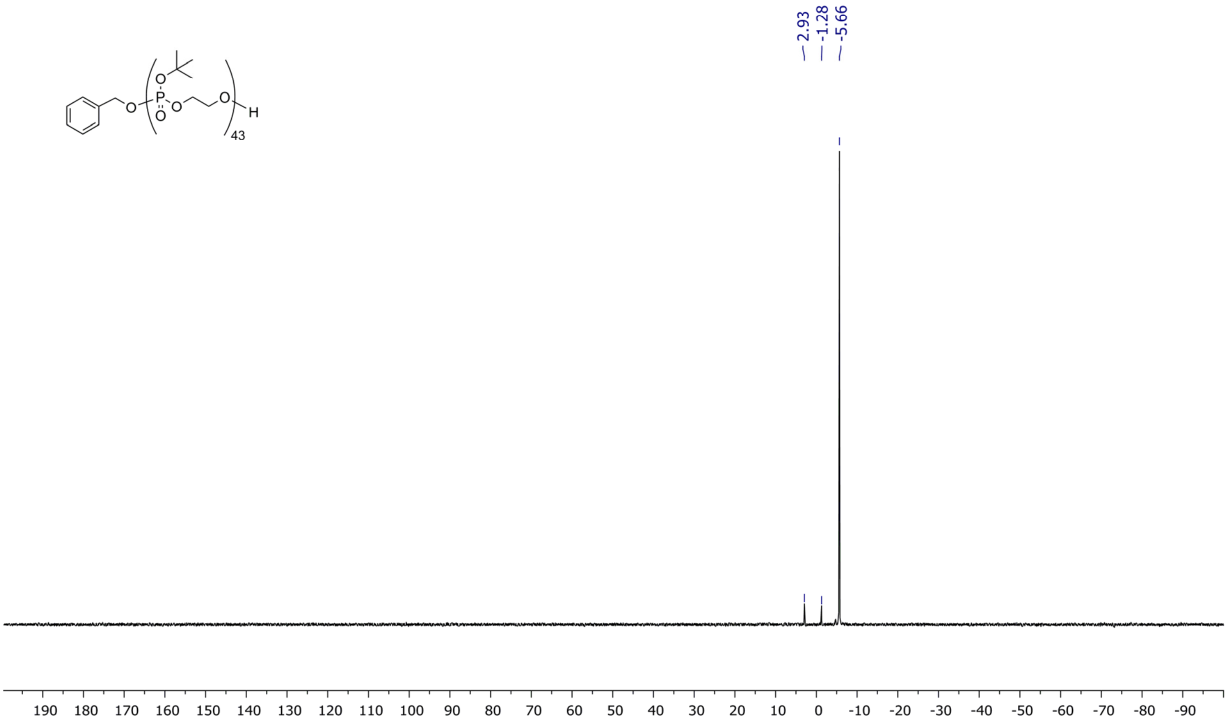

Figure S10. $^{31}P\{^{1}H\}$ NMR spectrum (162 MHz, $CDCl_3$, 20 °C) of poly(^{t}BuOEP).

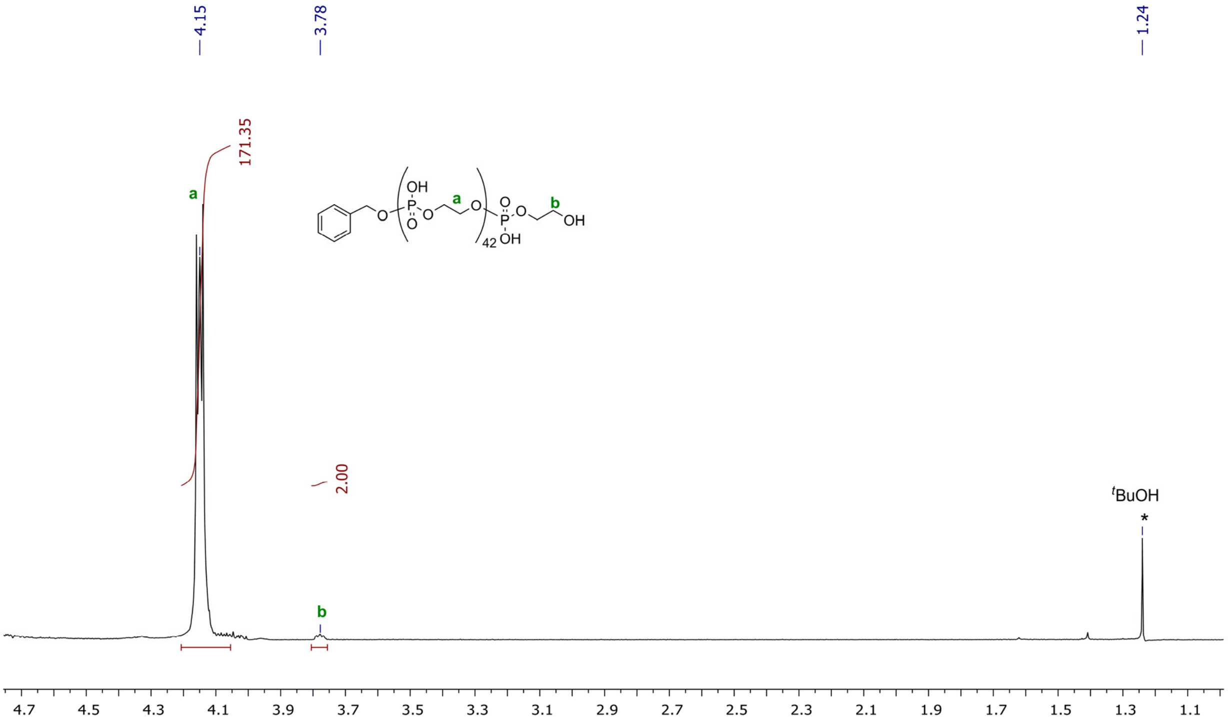

Figure S11. ^{1}H NMR spectrum (400 MHz, $CDCl_3$, 20 °C) of PEPA. The signals of ethylene phosphate fragment (a) and CH_2OH end-group (b) are highlighted.

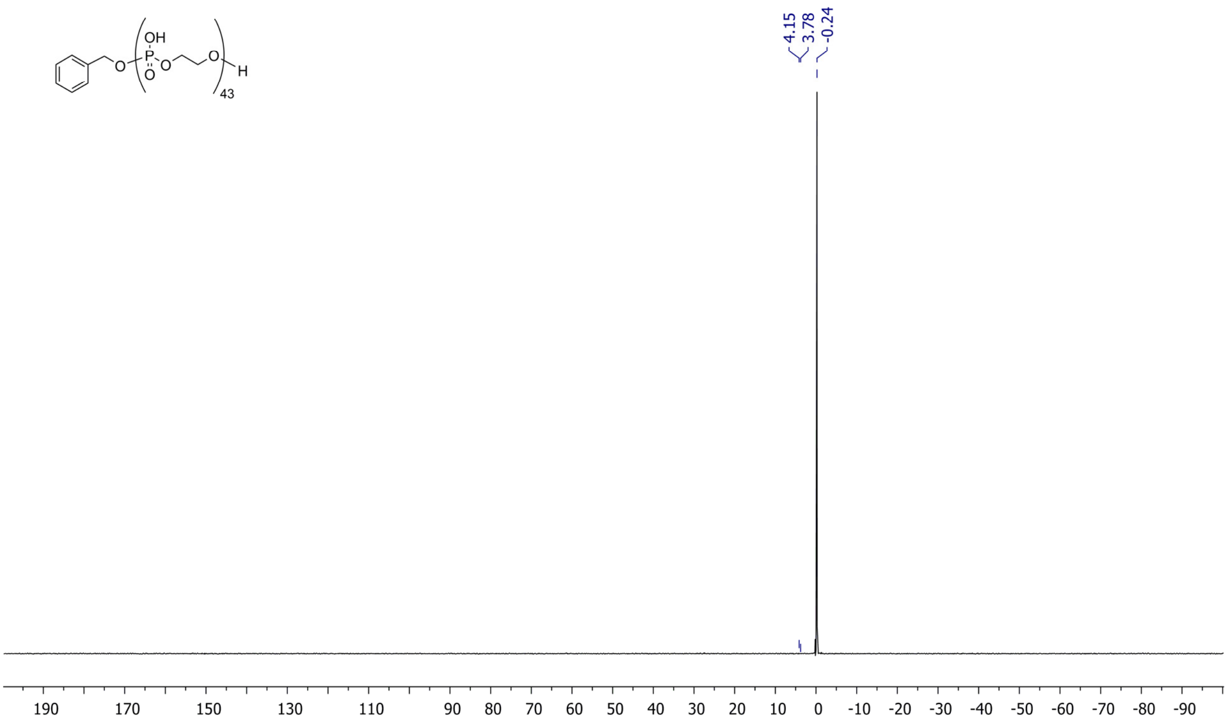

Figure S12. $^{31}P\{^{1}H\}$ NMR spectrum (162 MHz, $CDCl_3$, 20 °C) of PEPA.

^{31}P NMR spectroscopy was used for the determination of the concentration of phosphate groups in aqueous PEPA solution. Tromethyl phosphate (TMP, 27.9 mg) was added to 203 mg of PEPA solution. The resulting mixture was diluted by D_2O to total volume of 600 μl. The molar concentration of phosphate groups was determined by integration of the signals of TMP and PEPA in ^{31}P NMR spectrum of the mixture (Figure S13).

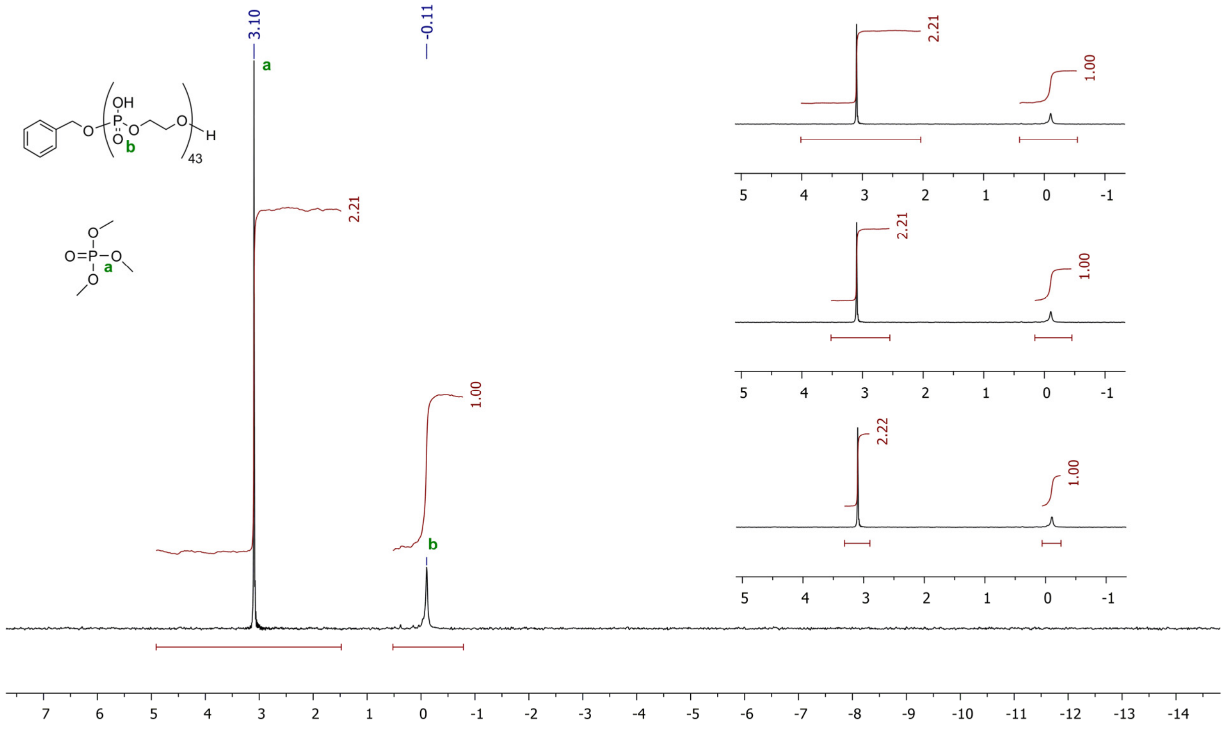

Figure S13. $^{31}P\{^{1}H\}$ NMR spectrum (162 MHz, $CDCl_3$, 20 °C) of TMP/PEPA mixture. The signals of phosphorus atoms in PEPA (a) and TMP (b) are highlighted.

S3. Study of cell proliferation

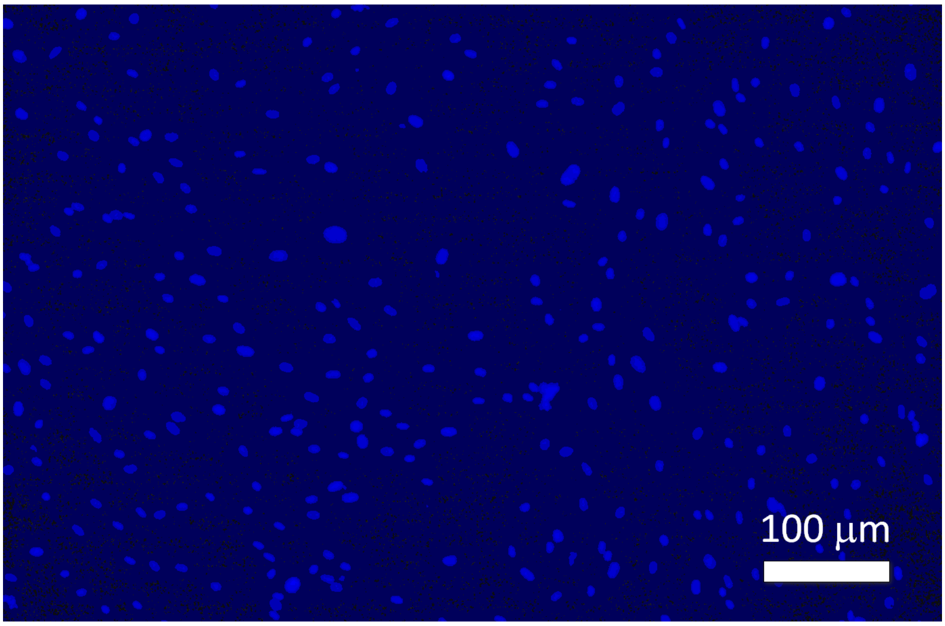

Figure S14. The microphotograph of DAPI-colored cells.

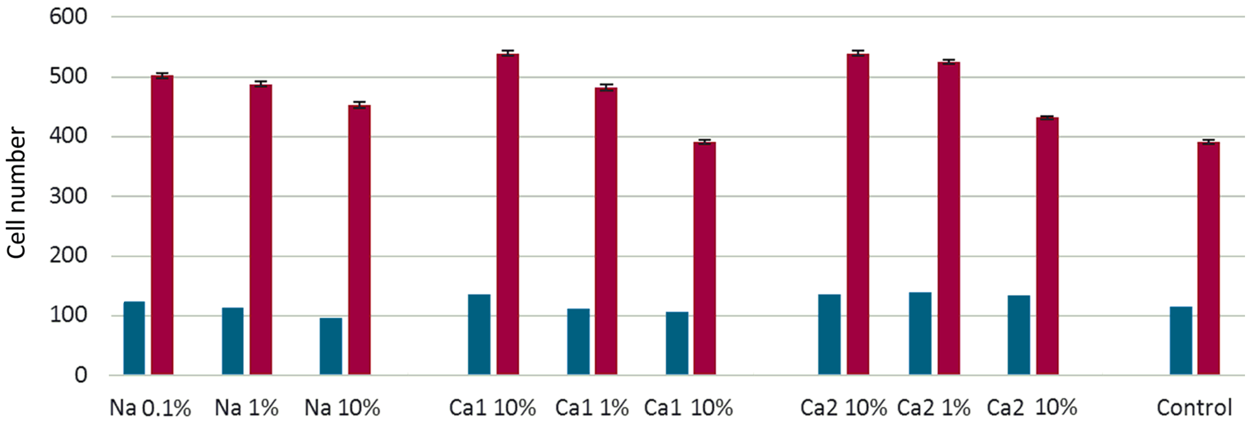

Figure S15. The results of cell adhesion and proliferation experiments for the solutions of PEPA metal salts (**Na**, **Ca1** and **Ca2**) diluted by the factors of 1000, 100 and 10. The numbers of cells in the field of view are presented in blue (after 1 day) and in red (after 7 days).
